# Functional status, pre-dialysis health and clinical outcomes among elderly dialysis patients

**DOI:** 10.1186/s12882-018-0898-1

**Published:** 2018-04-27

**Authors:** Silvi Shah, Anthony C. Leonard, Charuhas V. Thakar

**Affiliations:** 1 Division of Nephrology and Hypertension, University of Cincinnati, 231 Albert Sabin Way, Cincinnati, OH 45267 USA; 2 Department of Family and Community Medicine, University of Cincinnati, Cincinnati, OH USA; 3 Division of Nephrology, University of Cincinnati and VA Medical Center, Cincinnati, Ohio USA

**Keywords:** Functional status, Elderly, Mortality, Vascular access, and Dialysis

## Abstract

**Background:**

Elderly patients comprise the fastest growing population initiating dialysis in United States. The impact of poor functional status and pre-dialysis health status on clinical outcomes in elderly dialysis patients is not well studied.

**Methods:**

We studied a retrospective cohort of 49,645 incident end stage renal disease patients that initiated dialysis between January 1, 2008 and December 31, 2008 from the United States Renal Data System with linked Medicare data covering at least 2 years prior to dialysis initiation. Using logistic regression models adjusted for pre-dialysis health status and other cofounders, we examined the impact of poor functional status as defined from form 2728 on 1-year all-cause mortality as primary outcome, type of dialysis modality (hemodialysis vs. peritoneal dialysis), and type of initial vascular access (arteriovenous access vs. central venous catheter) among hemodialysis patients as secondary outcomes.

**Results:**

Mean age was 72 ± 11 years. At dialysis initiation, 18.7% reported poor functional status, 88.9% had at least 1 pre-dialysis hospitalization, and 27.8% did not receive pre-dialysis nephrology care. In adjusted analyses, 1-year mortality was higher in patients with poor functional status (OR, 1.48; 95% CI, 1.40–1.57). Adjusted odds of being initiated on hemodialysis than peritoneal dialysis (odds ratio [OR], 1.39; 95% confidence interval [CI], 1.16–1.66) were higher in patients with poor functional status. Poor functional status decreased the adjusted odds of starting hemodialysis with arteriovenous access as compared to central venous catheter (OR, 0.79; 95% CI, 0.72–0.86).

**Conclusion:**

Poor functional status in elderly patients with end stage renal disease is associated with higher odds of initiating hemodialysis; increases the risk of central venous catheter use, and is an independent predictor of 1-year mortality.

**Electronic supplementary material:**

The online version of this article (10.1186/s12882-018-0898-1) contains supplementary material, which is available to authorized users.

## Background

Elderly chronic kidney disease (CKD) patients defined as ≥60 years of age represent the fastest growing segment of the incident end stage renal disease (ESRD) population in United States, with more than half of incident dialysis patients being over 65 years of age [1]. The annual mortality in patients with ESRD is about 20%, and is higher in the first few months after the initiation of dialysis than after this period [2]. Older age is independently associated with a 5 fold increase in odds of death within 90 days of dialysis initiation [3]. Even though, early mortality is a significant problem among elderly ESRD patients, it remains under recognized [4]. Since most of the deaths occur within the first year of initiating dialysis, it is plausible that health conditions present prior and during dialysis initiation impact post-dialysis outcomes [2].

Activities of daily living are one of the most important prognostic factors that influence long-term outcomes [5, 6]. Functional status is commonly defined as an individual’s ability to perform normal activities of daily living required to meet basic needs, and maintain well-being [7]. Poor functional status is prevalent in older patients with chronic kidney disease, and is independently associated with a higher risk of death [8]. Comorbidities and acute care hospitalizations are frequent in elderly CKD patients, may lead to poor pre-dialysis health, and independently increase the risk of death [9–11]. Furthermore, initiation of dialysis is linked to decline in functional status in elderly patients with ESRD, and therefore can impact quality of life [6]. Although studies have examined clinical outcomes of elderly dialysis patients, the impact of functional status and pre-dialysis health including pre-dialysis acute hospitalization at incident dialysis on post-ESRD outcomes has not been studied.

The main objective of this study was to determine the impact of poor functional status on key clinical outcomes in elderly patients who get initiated on dialysis, after taking into account their pre-dialysis health including pre-dialysis acute hospitalizations. Using the most comprehensive national database of dialysis patients in United States, the United System Renal Data System (USRDS) with linked Medicare information, we hypothesized that poor functional status along with poor pre-dialysis health at the time of dialysis initiation is associated with higher mortality in ESRD patients. Additionally, we examined the impact of poor functional status with type of dialysis modality as well as type of vascular access during hemodialysis initiation.

## Methods

### Study population and study design

**Fig. 1:**
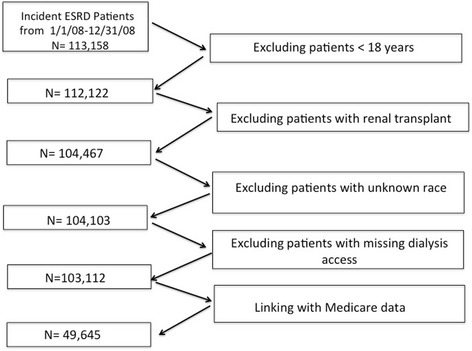
Flow chart describing the derivation of the study cohort

We included 49,645 patients ≥18 years, who initiated hemodialysis or peritoneal dialysis between January 1, 2008 and December 31, 2008 from the USRDS, and had both Medicare Part A and Part B coverage as their insurer at least 2 years prior to dialysis initiation. The cohort was predominantly older adults (≥ 60 years of age), and therefore hereby referred to as elderly in the present study. The data was obtained by a formal request to USRDS. The USRDS required the institutional review board to review the request, and the study was deemed exempt by University of Cincinnati institutional review board. We excluded patients < 18 years old and those with renal transplants. Patients with missing information on race and missing information on dialysis access were also excluded. Figure 1 shows the derivation of the study cohort.

### Exposure of interest

Our major predictor was poor functional status defined by any of the three co-morbid conditions as specified in form CMS-2728 – (1) inability to ambulate, (2) inability to transfer or (3) need of assistance with daily activities, as described in the prior studies [4, 12, 13].

### Primary and secondary outcomes

Primary outcome was 1-year all-cause mortality after the ESRD initiation date. Secondary outcomes examined were - type of dialysis modality (hemodialysis vs. peritoneal dialysis [PD]) and type of initial vascular access (arteriovenous [AV] access vs. central venous catheter [CVC]) among hemodialysis patients. Patients were followed from dialysis initiation to death, or censored at the end of our death records, December 31, 2010 (two to 3 years post-ESRD).

### Covariates

Form CMS-2728 was used to obtain information on dialysis modality, vascular access at hemodialysis initiation, ESRD cause, comorbidities (during the last 10 years), laboratory data, nursing home history, pre-dialysis nephrology care, and history of unemployment. For regression model analyses, we collapsed incident vascular access into two groups - AV access (including AV fistula and AV graft) vs. CVC (including others). We included the following covariates to determine the pre-dialysis health in patients with ESRD: pre-dialysis acute care hospitalizations, pre-dialysis nephrology care, nursing home residence, comorbidities, and laboratory data (serum hemoglobin and serum albumin within 45 days prior to most recent ESRD episode). Pre-ESRD Medicare claims was used to obtain information on acute hospitalizations during the 2 years preceding ESRD. The present analysis fell within the scope of the data acquisition, and institutional review board approval, whereby pre-ESRD information for up to 2 years prior to incident dialysis was available for the study period (January 1, 2008 and December 31, 2008), and therefore included in the study design. Comorbidities examined included congestive heart failure, atherosclerotic heart disease which included other cardiac disease, hypertension, diabetes mellitus, cancer, peripheral vascular disease, transient ischemic attack/cerebrovascular accident, chronic obstructive pulmonary disease and amputation [1].

### Statistical analyses

All data were analyzed using SAS (SAS Institute, Cary, NC). Summary statistics are presented as percentages for categorical data and mean ± standard deviation (SD) for continuous variables. Differences between groups were tested with chi-squared tests for categorical variables and t-tests or one-way ANOVAs for continuous variables. Multivariable logistic regression was used to derive the risk estimates from models, which included covariates, expressed as OR with 95% CI. Statistical significance was set at a two-tailed *p*-value of 0.05, unadjusted for multiple tests. Kaplan-Meier methods were used to construct unadjusted survival curves, and the survival rates of different groups were compared using a log-rank test. Using logistic regression models adjusted for pre-dialysis health status and other covariates, we examined the impact of poor functional status on hemodialysis vs. PD and in separate model among hemodialysis patients; we studied the effect of functional status on AV access vs. CVC. When using dialysis modality and access-type to predict death we used a 3-group covariate (PD, AV hemodialysis access, and CVC hemodialysis access). Predictor variables in the logistic regression models included demographics, comorbidities, laboratory data, ESRD cause, nursing home residence, unemployment history, transplant information provision, pre-ESRD nephrology care and pre-ESRD acute hospitalizations. Additionally, a Cox proportional hazard model, which included the same covariates as above, was used to study the impact of poor functional status on mortality. Finally, for purposes of examining its association with survival, we constructed a simple mortality risk score for each patient (ranging from zero to three), composed of patient’s count of three risk factors: poor functional status, octogenarian status, and history of any hospitalization during the 2 years pre-ESRD. Risk factors of functional status, octogenarian status and pre-dialysis hospitalization were chosen based on clinical relevance. Kaplan-Meier survival curves were estimated (1) comparing patients with and without poor functional status; and (2) comparing patients with four levels of risk scores.

## Results

### Baseline characteristics of study cohort

Clinical characteristics and demographics for the study population are shown in Table 1. The mean age was 72 ± 11 years. Of the study sample; 54.6% were male, 24.5% were Black, and 25.6% were octogenarian. With regards to comorbidities, 56.2% had diabetes, 85.2% had hypertension, 42.9% had atherosclerotic heart disease, 9.8% had history of malignancy, 3.5% had history of amputation, 18.1% had peripheral vascular disease, and 12.4% has history of transient ischemic attack/ cerebrovascular accident. 34.4% did not report being informed about kidney transplant options prior to initiating dialysis. Only 4.1% of patients initiated PD. Majority, 95% used dialysis facility as dialysis setting. Of those who started hemodialysis, 82.3% initiated with a CVC and the remaining 17.7% initiated with an AV access. Among pre-dialysis clinical factors, 51.8% had low serum albumin < 3.5 mg/dl, 68.3% had low hemoglobin < 11 g/dl, 88.9% had at least 1 hospitalization in the 2 years prior to initiation of dialysis, 10.3% had a history of a nursing home stay, and 27.8% had not seen a nephrologist prior to starting dialysis.

**Table 1:** Characteristics of patients who initiated dialysis in 2008 stratified by poor functional status and good functional status

Variable	All	Poor functional status	Good functional status	*P* value
Number of Subjects	49,645	9295 (18.7%)	40,350 (81.3%)	
Mortality, %				
90-Day	11.6	19.3	9.8	< 0.001
1-Year	31.2	46.4	27.7	< 0.001
Demographics				
Age (years)	72 (11)	73 (11)	71 (11)	< 0.001
18–29	0.2	0.2	0.2	< 0.001
30–39	1.2	0.8	1.2	
40–49	3.4	2.4	3.6	
50–59	9.1	8.3	9.3	
60–69	23.6	21.1	24.2	
70–79	36.9	35.3	37.3	
80–89	23.6	28.9	22.4	
90–100	2.0	3.0	1.8	
Octogenarians, %	25.6	31.9	24.2	< 0.001
Body mass index (kg/m^2^)	28.7 (7.7)	28.9 (8.4)	28.6 (7.5)	0.002
< 18.5	3.7	4.9	3.4	< 0.001
18.5–24.99	31.8	32.0	31.8	
25–30	28.5	25.4	29.3	
> 30	34.4	35.8	34.0	
Missing	1.6	1.9	1.6	
Male, %	54.6	48.8	55.9	< 0.001
Race, %				0.59
Hispanic	9.3	9.7	9.2	
White	62.3	61.8	62.4	
Black	24.5	24.6	24.5	
Asian	3.0	2.9	3.0	
Native American	0.9	1.0	0.9	
Comorbidities, %				
Congestive heart failure	40.8	52.8	38.0	< 0.001
ASHD	42.9	53.6	40.4	< 0.001
Hypertension	85.2	86.5	84.9	< 0.001
Diabetes mellitus	56.2	63.4	54.5	< 0.001
Cancer	9.8	10.7	9.5	< 0.001
Amputation	3.5	8.1	2.5	< 0.001
Peripheral vascular disease	18.1	26.4	16.2	< 0.001
TIA/CVA	12.4	21.0	10.4	< 0.001
COPD	12.6	18.7	11.2	< 0.001
Dialysis modality, %				
Hemodialysis	95.9	98.4	95.3	< 0.001
Peritoneal dialysis	4.1	1.6	4.7	
Hemodialysis access, %				
AV access	17.7	10.7	19.3	< 0.001
Central venous catheter	82.3	89.3	80.7	
Maturing AV access (among CVC)	21.4	17.2	22.4	< 0.001
Laboratory variables from within 45-day period prior to most recent ESRD episode				
Albumin, g/dL	3.1 (0.7)	2.9 (0.7)	3.2 (0.7)	< 0.001
< 3.5	51.8	60.5	49.7	< 0.001
≥ 3.5	25.3	16.8	27.2	
Missing	23.0	22.7	23.0	
Hemoglobin, g/dL	10.0 (1.5)	9.9 (1.5)	10.1 (1.5)	< 0.001
< 11	68.3	71.8	67.5	< 0.001
11–12	15.1	13.3	15.5	
> 12	8.5	8.0	8.6	
Missing	8.1	6.9	8.4	
Dialysis setting, %				
Dialysis facility	95.0	95.7	94.8	< 0.001
Home	4.3	1.9	4.8	
Skilled nursing facility	0.7	2.3	0.4	
Employment status, %				
Unemployed	11.1	10.5	11.2	< 0.001
Employed + others	5.9	5.0	6.1	
Retired-age	61.6	61.0	61.7	
Retired-disability	21.4	23.5	20.9	
Acute hospitalization during 2- year pre-ESRD				
Frequency, %	88.9	93.8	87.8	< 0.001
Total length of stay (days)	23 (28)	36 (36)	20 (25)	< 0.001
Pre-dialysis nephrology care, %				
None	27.8	35.3	26.1	< 0.001
< 12 months	33.0	28.2	34.2	
> 12 months	26.3	21.3	27.5	
Unknown	12.8	15.1	12.3	
Cause of end stage renal disease, %				
Diabetes mellitus	45.9	49.7	45.1	< 0.001
Hypertension/LVD	32.3	29.9	32.8	
Malignancy	2.6	2.0	2.8	
Cystic/hereditary	1.3	0.6	1.4	
Secondary GN /vasculits	1.2	0.9	1.3	
Glomerulonephritis	3.9	2.2	4.3	
Interstitial nephritis/pyelonephritis	2.9	2.8	2.9	
Others	9.8	11.8	9.3	
Cause of death (among patients with 1-year mortality), %				
Cardiovascular	38.2	37.1	38.7	< 0.001
Infection	10.0	12.1	9.2	
Malignancy	4.5	3.1	5.0	
Withdrawal of dialysis	11.6	11.6	11.7	
Unknown/others	35.6	36.1	35.4	
History of nursing home, %	10.3	38.2	3.9	< 0.001
Provision of transplant information, %	65.6	55.5	67.9	< 0.001

Data are presented in mean (*SD*) or proportion where appropriate. *CVA/TIA* cerebrovascular accident/transient ischemic attack, *COPD* chronic obstructive pulmonary disease, *AV* arteriovenous, *CVC* central venous catheter, *LVD* large vessel disease, *GN* glomerulonephritis

At dialysis initiation, 18.7% of patients were reported to have history of poor functional status. Patients with poor functional status were more likely to be octogenarians (31.9% vs. 24.2%, *p* < 0.001), females (51.2% vs. 44.1%, *p* < 0.001), and nursing home residents (38.2% vs. 3.9%, *p* < 0.001). Patient with poor functional status also had higher rates of other comorbidities, and low serum hemoglobin (71.8% vs. 67.5%, *p* < 0.001) or low serum albumin (60.5% vs. 49.7%, *p* < 0.001). Furthermore, patients with poor functional status were provided with lower frequency of transplant information prior to initiating dialysis (55.5% vs. 67.9%, *p* < 0.001). Examination of pre-dialysis clinical factors revealed that higher frequency of patients with poor functional status initiated dialysis without any prior nephrology care (35.3% vs. 26.1%, *p* < 0.001). Pre-dialysis hospitalizations were associated with poor functional status (93.8% vs. 87.8%, *p* < 0.001). Furthermore, total hospital days stay during 2-year pre-dialysis hospitalizations was longer in patients with poor functional status (35.6 days vs. 20.4 days, *p* < 0.001).

### Primary and secondary outcomes

Overall, 1-year all cause mortality was 31.2% and 90-day mortality was 11.6%. Among the deceased patients, death from a cardiovascular cause (38.2%) and withdrawal of dialysis (11.6%) were the most common causes of death. Patients with poor functional status had higher unadjusted one-year mortality than those with good functional status (46.4% vs. 27.7%, *p* < 0.001). Table 2 shows the patient characteristics associated with 1-year mortality. Patients with one-year mortality more frequently lacked pre-ESRD nephrology care (35% vs. 24.5%, *p* < 0.001), resided more often in a nursing home (18% vs. 6.8%, *p* < 0.001), had higher rates of pre-dialysis hospitalization (93.4% vs. 86.9%, *p* < 0.001) and were less likely to be provided with transplant information (57.9% vs. 69.1%, *p* < 0.001).

**Table 2:** Characteristics of patients who initiated dialysis in 2008 stratified by 1-year mortality

Variable	All	Death ≤ 1 year	Death > 1 year	*P* value
Number of Subjects	49,645	15,513 (31.2%)	34,132 (68.8%)	
Functional status, %				
Poor functional status	18.7	27.8	14.6	< 0.001
Demographics				
Age (years)	72 (11)	75 (10)	70 (12)	< 0.001
18–29	0.2	0.1	0.3	< 0.001
30–39	1.2	0.5	1.4	
40–49	3.4	1.7	4.2	
50–59	9.1	5.7	10.6	
60–69	23.6	18.2	26.1	
70–79	36.9	38.1	36.3	
80–89	23.6	32.1	19.7	
90–100	2.0	3.4	1.4	
Octogenarians, %	25.6	35.6	21.1	< 0.001
Body mass index, kg/m^2^	28.7 (7.7)	7.5	29.1 (7.7)	< 0.001
< 18.5	3.7	5.2	3.0	< 0.001
18.5–24.99	31.8	36.5	29.7	
25–30	28.5	27.2	29.1	
> 30	34.4	29.5	36.6	
Missing	1.6	1.7	1.6	
Male, %	54.6	55.6	54.1	0.003
Race, %				< 0.001
Hispanic	9.3	7.1	10.3	
White	62.3	70.2	58.7	
Black	24.5	19.5	26.7	
Asian	3.0	2.3	3.3	
Native American	0.9	0.8	1.0	
Comorbidities, %				
Congestive heart failure	40.8	49.1	37.0	< 0.001
ASHD	42.9	48.7	40.2	< 0.001
Hypertension	85.2	81.3	87.0	< 0.001
Diabetes mellitus	56.2	52.4	57.9	< 0.001
Cancer	9.8	12.5	8.5	< 0.001
Amputations	3.5	3.8	3.4	0.03
Peripheral vascular disease	18.1	20.7	16.9	< 0.001
TIA/CVA	12.4	14.0	11.6	< 0.001
COPD	12.6	16.8	10.7	< 0.001
Dialysis modality, %				
Hemodialysis	95.9	97.7	95.0	< 0.001
Peritoneal dialysis	4.1	2.3	5.0	
Hemodialysis access, %				
AV access	17.7	9.1	21.6	< 0.001
Central venous catheter	82.3	90.9	78.4	
Maturing AV access (among CVC)	21.4	15.1	24.7	< 0.001
Laboratory variables from within 45-day period prior to most recent ESRD episode				
Albumin, g/dL	3.1 (0.7)	3.0 (0.7)	3.2 (0.7)	< 0.001
< 3.5	51.8	57.6	49.1	
≥ 3.5	25.3	18.4	28.4	< 0.001
Missing	23.0	24.0	22.5	
Hemoglobin, g/dL	10.0 (1.5)	10.0 (1.5)	10.1 (1.5)	
< 11	68.3	69.1	67.9	
11–12	15.1	13.9	15.7	
> 12	8.5	8.3	8.6	0.05
Missing	8.1	8.7	7.8	< 0.001
Dialysis setting, %				
Dialysis facility	95.0	96.2	94.5	< 0.001
Home	4.3	2.5	5.1	
Skilled nursing facility	0.7	1.3	0.5	
Employment status, %				
Unemployed	11.1	10.2	11.5	< 0.001
Employed + others	5.9	4.7	6.4	
Retired-age	61.6	68.3	58.5	
Retired-disability	21.4	16.7	23.5	
Acute hospitalization during 2 year Pre-ESRD				
Frequency, %	88.9	93.4	86.9	< 0.001
Total length of stay (days)	23.3 (27.9)	31.1 (32.3)	19.7 (24.9)	< 0.001
Pre-dialysis nephrology care, %				
None	27.8	35.0	24.5	< 0.001
< 12 months	33.0	29.3	34.8	
> 12 months	26.3	20.0	29.2	
Unknown	12.8	15.7	11.5	
Cause of end stage renal disease, %				
Diabetes mellitus	45.9	40.8	48.3	< 0.001
Hypertension/LVD	32.3	34.1	31.5	
Malignancy	2.6	4.2	1.9	
Cystic/hereditary	1.3	0.7	1.5	
Secondary GN/vasculits	1.2	1.1	1.3	
Glomerulonephritis	3.9	2.8	4.4	
Interstitial nephritis/pyelonephritis	2.9	2.9	2.8	
Others	9.8	13.3	8.2	
History of nursing home, %	10.3	18.0	6.8	< 0.001
Provision of transplant information, %	65.6	57.9	69.1	< 0.001

Data are presented in mean (*SD*), or proportion where appropriate. *CVA/TIA* cerebrovascular accident/transient ischemic attack, *COPD* chronic obstructive pulmonary disease, *AV* arteriovenous, *CVC* central venous catheter, *LVD* large vessel disease, *GN* glomerulonephritis

**Fig. 2:**
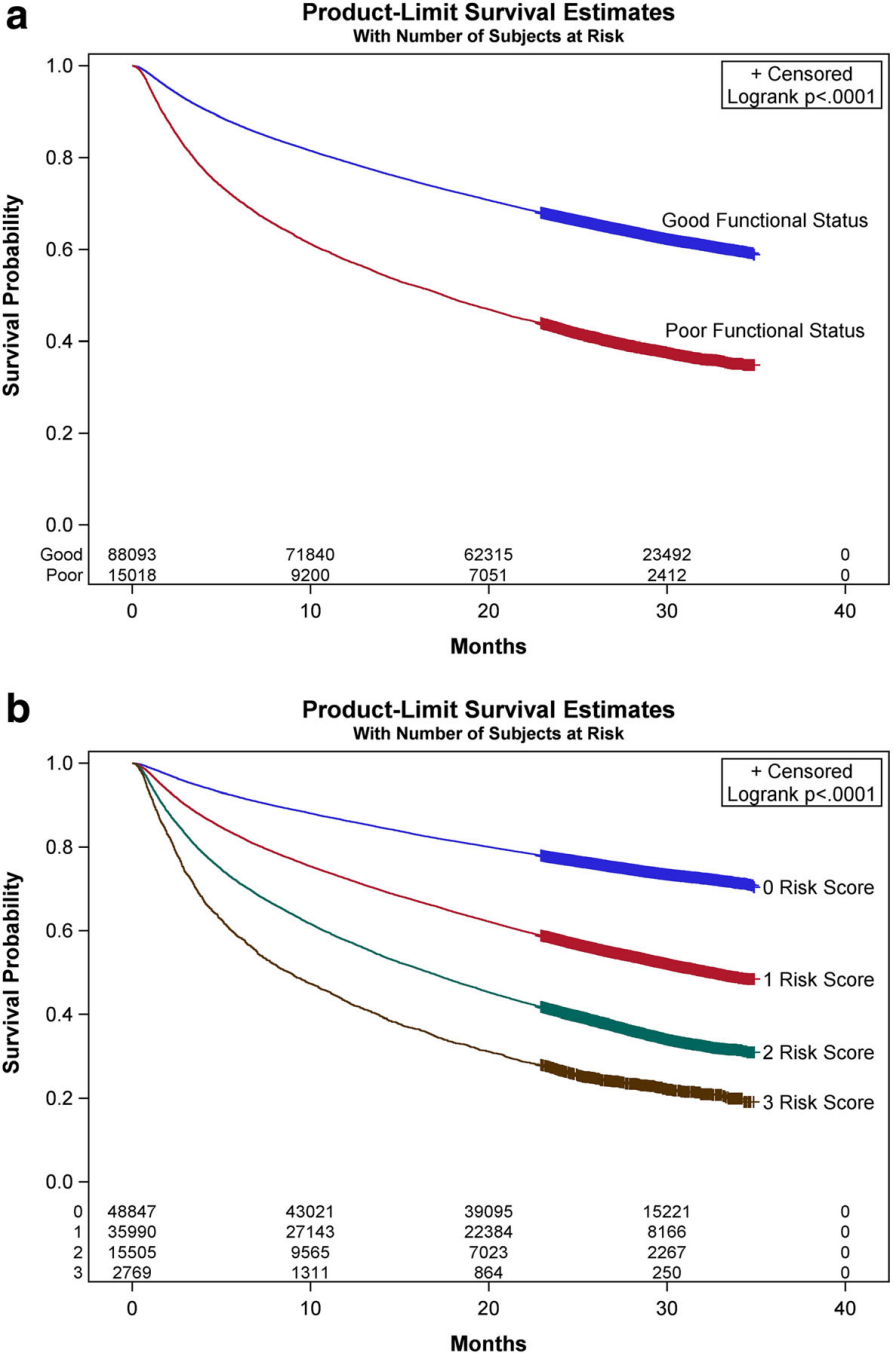
Kaplan-Meier survival curves by end stage renal disease to death in dialysis patients by (**a**) functional status, and (**b**) risk score levels

**Fig. 3:**
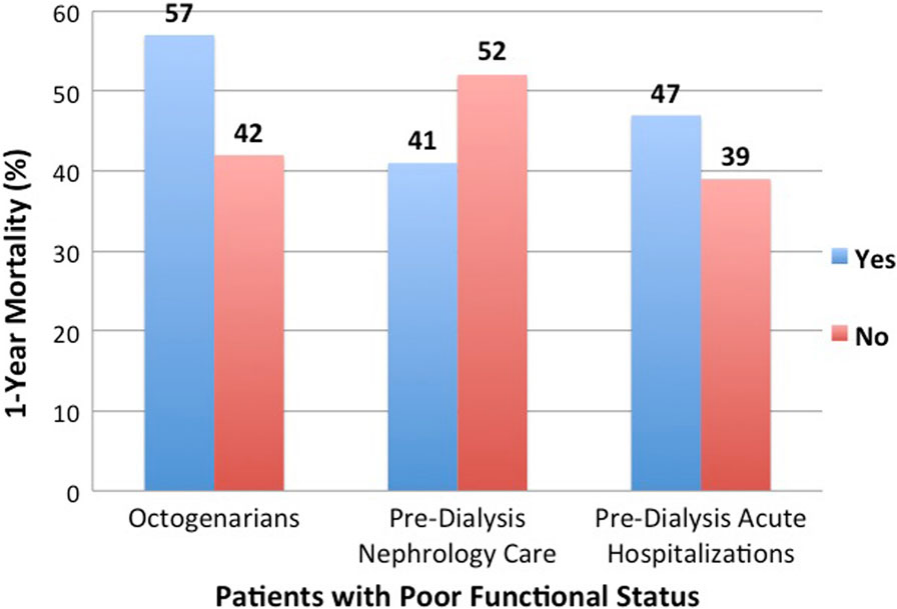
One-year mortality in incident end stage renal disease patients with poor functional status by octogenarian status, pre-dialysis nephrology care and pre-dialysis acute care hospitalization

Figure 2a shows Kaplan-Meier survival curves comparing patients with and without poor functional status. In this time-to-event analysis, poor functional status was associated with lower survival (log-rank test *p* < 0.0001). Figure 2b represents Kaplan-Meier survival analysis based on level of the risk scores depending on absence or presence of poor functional status, octogenarian status and pre-ESRD hospitalizations (log rank test *p* < 0.0001). One-year mortality at the different levels of our risk score was 14%, 26%, 42%, and 57% for scores of 0, 1, 2, and 3 respectively. Figure 3 shows rates of one-year mortality among those with poor functional status by octogenarian status, pre-dialysis nephrology care and acute care hospitalization. Of note, octogenarians with poor functional status had 1-year mortality rate of 57% compared to 42% in those less than 80 years of age (*p* value < 0.001). However, the interaction between poor functional status and octogenarians for the outcome of one-year mortality in patients with ESRD was not significant (*p* = 0.15). By multivariate analysis, one-year adjusted mortality was higher in patients with poor functional status (odds ratio [OR], 1.48; 95% confidence interval [CI], 1.40–1.57) (Table 3). In Cox proportional hazard model (Additional file 1), 51.3% of the study cohort patients died during a mean follow up period of 21.7 ± 10.6 months; and poor functional status was associated with higher risk of death (hazard ratio [HR], 1.28 (1.24–1.33), *p* < 0.001).

**Table 3:** Independent variable predictors of 1-year mortality in incident end stage renal disease patients in the final regression model

Variable^a^	Odds ratio (95% CI)
Poor functional status	1.48 (1.40, 1.56)
Dialysis access	
Central venous catheter	1.84 (1.72, 1.97)
Peritoneal dialysis	1.24 (1.08, 1.42)
Age ≥ 80 years	1.65 (1.57, 1.73)
Body mass index, kg/m^2^	
< 18.5	1.82 (1.64, 2.03)
18.5–25	1.34 (1.27, 1.41)
25–30	1.10 (1.04, 1.16)
Missing	1.19 (1.01, 1.40)
Females	0.94 (0.91, 0.99)
Race	
Asians	0.63 (0.55, 0.71)
Blacks	0.68 (0.65, 0.72)
Hispanics	0.65 (0.60, 0.70)
Native Americans	0.83 (0.67, 1.03)
History of nursing home	1.80 (1.67, 1.92)
Unemployment	1.04 (0.98, 1.12)
Comorbidities	
Congestive heart failure	1.37 (1.32, 1.44)
Atherosclerotic heart disease	1.11 (1.07, 1.16)
Hypertension/large vessel disease	0.72 (0.68, 0.77)
Diabetes mellitus	0.97 (0.92, 1.03)
Cancer	1.27 (1.19, 1.36)
Amputation	0.98 (0.88, 1.10)
Peripheral vascular disease	1.10 (1.04, 1.16)
CVA/TIA	1.06 (1.00, 1.13)
Chronic obstructive pulmonary disease	1.29 (1.22, 1.37)
Albumin, g/dL	
< 3.5	1.42 (1.35, 1.50)
Missing	1.33 (1.25, 1.41)
Hemoglobin, g/dL	
< 11	0.93 (0.87, 1.01)
11–12	0.90 (0.83, 0.99)
Missing	1.06 (0.96, 1.17)
Pre-dialysis acute hospitalization	1.31 (1.21, 1.42)
Cause of ESRD	
Cystic/hereditary	0.71 (0.57, 0.88)
Glomerulonephritis	0.76 (0.68, 0.86)
Hypertension/large vessel disease	1.08 (1.02, 1.14)
Interstitial nephritis/pyelonephritis	0.97 (0.85, 1.10)
Others/unknown	1.26 (1.17, 1.36)
Malignancy	2.07 (1.82, 2.35)
Vasculits/secondary GN	0.96 (0.79, 1.16)
Pre-dialysis nephrology care	
0–12 months	0.78 (0.74, 0.82)
> 12 months	0.70 (0.66, 0.74)
Unknown	1.05 (0.99, 1.12)
Access to transplant information	0.81 (0.77, 0.84)

*ESRD* end stage renal disease, *CVA/TIA* cerebrovascular accident/transient ischemic attack, *GN* glomerulonephritis

^a^Referents were good functional status to poor functional status, arteriovenous access for vascular access, > 30 for body mass index, male for females, White for race, no for comorbidities, > 3.5 mg/dl for serum albumin, > 12 for serum hemoglobin,, and diabetes mellitus for ESRD cause

**Fig. 4:**
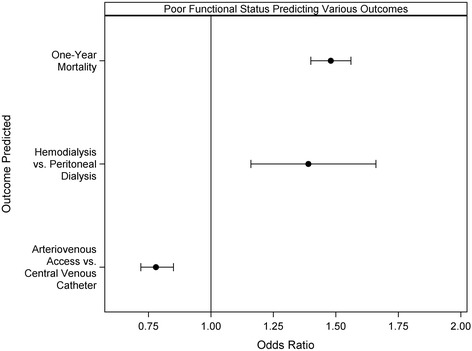
Association of poor functional status with outcomes of one-year mortality, type of dialysis modality (hemodialysis vs. peritoneal dialysis), and type of vascular access (arteriovenous access vs. central venous catheter) for hemodialysis initiation

For the outcome of dialysis modality, patients with poor functional status had higher use of hemodialysis as the initial dialysis modality than those with good functional status (98.4% vs. 95.3%, *p* < 0.001), an association that was maintained in the adjusted models (OR, 1.39; 95% CI, 1.16–1.66 (Table 4). Among those that initiated hemodialysis with CVC, poor functional status was associated with lower rates of maturing AV access (15.1% vs. 24.7%, p < 0.001). AV access was the less frequently used hemodialysis access in those with poor functional status (10.7% vs. 19.3%, *p* < 0.001). In adjusted analyses, patients with poor functional status had lower odds of initiating dialysis with AV access than patients with good functional status (OR, 0.79; CI, 0.72–0.86) (Table 5). Figure 4 shows the association of poor functional status with outcomes of 1-year mortality, type of dialysis modality, and type of vascular access for hemodialysis initiation.

**Table 4:** Independent variable predictors of hemodialysis vs. peritoneal dialysis as the dialysis modality in the final regression model

Variable^a^	Odds ratio (95% CI)
Poor functional status	1.39 (1.16, 1.66)
Age ≥ 80 years	1.70 (1.50, 1.94)
Body mass index, kg/m^2^	
< 18.5	0.92 (0.81, 1.04)
18.5–25	0.85 (0.76, 0.96)
25–30	1.27 (0.93, 1.75)
Missing	0.88 (0.60, 1.29)
Females	0.93 (0.85, 1.02)
Race	
Asians	1.15 (0.90, 1.48)
Blacks	1.88 (1.65, 2.14)
Hispanics	1.56 (1.30, 1.88)
Native Americans	0.94 (0.60, 1.45)
History of nursing home	3.86 (2.54, 5.85)
Unemployment	1.09 (0.93, 1.28)
Comorbidities	
Congestive heart failure	1.35 (1.21, 1.51)
Atherosclerotic heart disease	1.01 (0.91, 1.12)
Hypertension	0.96 (0.84, 1.11)
Diabetes mellitus	0.97 (0.86, 1.11)
Cancer	1.01 (0.86, 1.89)
Peripheral vascular disease	1.08 (0.95, 1.24)
CVA/TIA	0.95 (0.81, 1.10)
Chronic obstructive pulmonary disease	1.40 (1.18, 1.67)
Albumin, g/dL	
< 3.5	2.15 (1.93, 2.40)
Missing	1.42 (1.25, 1.61)
Hemoglobin, g/dL	
< 11	1.86 (1.63, 2.14)
11–12	1.16 (0.99, 1.36)
Missing	1.49 (1.21, 1.83)
Pre-dialysis acute hospitalization	2.61 (2.35, 2.90)
Cause of ESRD	
Cystic/hereditary	0.82 (0.61, 1.10)
Glomerulonephritis	0.61 (0.50, 0.75)
Hypertension/large vessel disease	0.92 (0.80, 1.05)
Interstitial nephritis/pyelonephritis	1.19 (0.88, 1.60)
Others/unknown	1.15 (0.92, 1.43)
Malignancy	1.05 (0.75, 1.47)
Vasculits/secondary GN	0.83 (0.55, 1.25)
Pre-dialysis nephrology care	
0–12 months	0.23 (0.19, 0.28)
> 12 months	0.23 (0.19, 0.28)
Unknown	0.67 (0.52, 0.88)
Access to transplant information	0.50 (0.44, 0.56)

*ESRD* end stage renal disease, *CVA/TIA* cerebrovascular accident/transient ischemic attack, *GN* glomerulonephritis

^a^Referents were good functional status to poor functional status, > 30 for body mass index, White for race, males for females, no for comorbidities, > 3.5 mg/dl for serum albumin, > 12 for serum hemoglobin,, and diabetes mellitus for ESRD cause

**Table 5:** Independent variable predictors of arteriovenous access vs. central venous catheter as the vascular access for hemodialysis initiation in the final regression model

Variable^a^	Odds ratio (95% CI)
Poor functional status	0.79 (0.72, 0.86)
Age ≥ 80 years	0.88 (0.82, 0.93)
Body mass index, kg/m^2^	
< 18.5	0.96 (0.82, 1.11)
18.5–25	0.97 (0.91, 1.04)
25–30	1.03 (0.96, 1.10)
Missing	1.02 (0.83, 1.27)
Females	0.83 (0.79, 0.88)
Race	
Asians	0.94 (0.81, 1.10)
Blacks	1.10 (1.03, 1.17)
Hispanics	0.81 (0.74, 0.90)
Native Americans	0.88 (0.67, 1.17)
History of nursing home	0.71 (0.63, 0.80)
Unemployment	1.06 (0.98, 1.16)
Comorbidities	
Congestive heart failure	0.75 (0.71, 0.80)
Atherosclerotic heart disease	0.92 (0.87, 0.97)
Hypertension	1.18 (1.09, 1.28)
Diabetes mellitus	0.96 (0.89, 1.03)
Cancer	0.97 (0.87, 1.06)
Amputation	0.88 (0.75, 1.03)
Peripheral vascular disease	1.02 (0.95, 1.09)
CVA/TIA	1.36 (0.95, 1.12)
Chronic obstructive pulmonary disease	0.87 (0.79, 0.94)
Albumin, g/dL	
< 3.5	0.52 (0.49, 0.55)
Missing	0.58 (0.54, 0.63)
Hemoglobin, g/dL	
< 11	0.87 (0.79, 0.95)
11–12	1.18 (1.06, 1.31)
Missing	0.99 (0.87, 1.12)
Pre-dialysis acute hospitalization	0.41 (0.38, 0.44)
Cause of ESRD	
Cystic/hereditary	1.40 (1.11, 1.66)
Glomerulonephritis	0.93 (0.82, 1.07)
Hypertension/large vessel disease	0.88 (0.82, 0.95)
Interstitial nephritis/pyelonephritis	0.83 (0.70, 0.98)
Others/unknown	0.52 (0.45, 0.59)
Malignancy	0.56 (0.46, 0.69)
Vasculits/secondary GN	0.52 (0.39, 0.70)
Pre-dialysis nephrology care	
0–12 months	6.49 (5.86, 7.19)
> 12 months	11.42 (10.31, 12.65)
Unknown	1.77 (1.54, 2.04)
Access to transplant information	1.18 (1.12, 1.25)

*ESRD* end stage renal disease, *CVA/TIA* cerebrovascular accident/transient ischemic attack, *GN* glomerulonephritis

^a^Referents were good functional status to poor functional status, arteriovenous access for vascular access, > 30 for body mass index, male for gender, White for race, no for comorbidities, > 3.5 mg/dl for serum albumin, > 12 for serum hemoglobin,, and diabetes mellitus for ESRD cause

## Discussion

In this national sample, we found that one-in-five patients who initiated dialysis did so with poor functional status, and when present and after taking pre-dialysis health into account, it was associated with higher mortality, lower likelihood of PD and greater use of CVC for hemodialysis initiation. To our knowledge, this is among the first reports to have examined the effect of functional status and pre-dialysis health status including pre-dialysis hospitalizations on long-term dialysis outcomes among older adults in United States.

There is a high prevalence of disability in elderly hemodialysis patients and observational studies have reported higher mortality with poor functional status in established dialysis patients [14, 15]. Kurella et al. studied the trajectory of functional status before and after the initiation of dialysis among elderly nursing home residents with ESRD. The authors concluded that initiation of dialysis in elderly nursing home residents was associated with substantial decline in functional status and worsening quality of life, with more than half dying at 1-year after dialysis initiation [6]. In another study, severely and moderately impaired functional status was significantly associated with early death after initiating dialysis (adjusted risk ratios: 3.93 and 2.38, respectively) [15]. However, none of these studies took into account pre-dialysis health status and health care prior to dialysis initiation.

The elderly CKD population in United States has a unique medical and social profile, and represents the fastest growing population in Unites States with ESRD [10]. Elderly patients initiating dialysis have a high prevalence of comorbidities that is associated with poor survival [16]. Pre-dialysis hospitalizations and lack of pre-ESRD nephrology care can further impact long-term outcomes in them [17]. Some retrospective studies have looked at the impact of pre-dialysis factors in isolation on mortality among older adults. In a German hemodialysis center, later referral to a nephrologist was associated with 1.8 fold increase in mortality in those over 75 years of age [18]. Crews et al. studied the association of pre-dialysis health status with focus on timing of dialysis initiation among older adults in United States. They concluded that early initiators were more likely to have all cause hospitalization days preceding dialysis initiation; and that early dialysis initiation did not benefit patients with functional limitation [12]. The present study is unique since it examined the impact of poor functional status at dialysis initiation along with pre-dialysis health status on post dialysis outcomes. In our study, one-fifth of the elderly patients were reported to have poor functional status at the time of dialysis initiation and by the end of 12 months, about one-third of them had died. Older patients with poor functional status were also associated with higher odds of pre-dialysis hospitalizations, and were less likely to receive nephrology care prior to initiation dialysis.

Concerns surrounding poor functional status are complex, and are difficult to modify once dialysis begins [6]. Functional status and comorbid disease burden in elderly patients with advanced chronic kidney disease referred for pre-dialysis education are independent predictors of mortality [8, 19]. Our analysis allows the opportunity to consider functional status prior to dialysis initiation along with pre-dialysis health status. To that effect, we specifically looked at the risk factors of lack of pre-dialysis nephrology care, presence of pre-dialysis acute care hospitalizations, comorbidities, and octogenarian status. This information on clinical outcomes in elderly dialysis patients becomes very important, since pre-dialysis health status is usually not taken into account when older adults are initiated on dialysis. Thamer et al. developed a shared decision making tool for nephrologists and patients to estimate the risk of early mortality following initiation of dialysis. Older age, low albumin, assistance with daily activities, nursing home history, comorbidities of cancer and heart failure; and hospitalizations prior to initiating dialysis were the 7 significant predictors of mortality. However, using their model, which was different from ours, mortality could only be predicted at 3 months and 6 months but not at 1 year [4]. Our study concludes that poor functional status in elderly dialysis patients, when adjusted for lack of pre-dialysis nephrology care, pre-dialysis acute hospitalizations, comorbidities and other covariates has a significant impact on 1-year mortality; and increases the risk of dying within 1 year by 48%. It therefore becomes imperative for clinicians to incorporate screening strategies for functional impairment with their routine plan of care. Patients who are identified to be at risk for functional decline may benefit from referral to a geriatrician, interdisciplinary care and physical rehabilitation [20]. Future prospective studies are, however, required to study the impact of the physical rehabilitation on long-term outcomes in elderly dialysis patients.

Several retrospective studies have evaluated vascular access outcomes in the elderly, but none of them have taken functional status and pre-dialysis health into account [21, 22]. Xue et al. reported that more than half of elderly patients had CVC at dialysis initiation with hazard ratio of 1.7 for 1-year mortality as compared to those with AVF [23]. In our study, more than two thirds of elderly patients started hemodialysis with a CVC; and had 35% chance of dying before 1 year. Additionally, patients with poor functional status who used CVC for hemodialysis initiation had 1-year mortality of 49%. While there are no uniform guidelines for access planning in the patients over 60-years of age, functional status prior to dialysis and not age alone may need to be considered in guiding our decisions regarding ideal vascular access placement in elderly patients. Observational studies have reported similar survival and quality of life in elderly patients undergoing hemodialysis vs. peritoneal dialysis [24, 25]. Even though, peritoneal dialysis modality may be a better option in elderly patients due to lack of vascular access, gentle ultrafiltration and ease of performing at home; its use may be limited among those with poor functional status [25, 26]. With assistance required in the daily activities, it may be therefore more appropriate for elderly ESRD patients with poor functional to be initiated on hemodialysis than peritoneal dialysis; and utilize CVC than AV access for hemodialysis initiation, similar to the findings seen in the present study.

Our study has limitations. Observational study and administrative information limit the information to be associative and cannot establish causality. Data from USRDS used for the study was 10 years old and there is a possibility of changes in the patient characteristics and patient care over the subsequent years. However, our report utilizes the information from the largest administrative database for dialysis patients that are maintained prospectively in United States. Functional status was defined by the reporting of physicians on form 2728, which can introduce misclassification bias and recall bias. Even though characterization of functional status occurred only once during initiation of dialysis in form 2728, and could change over time, this may provide physicians a quick assessment of individual’s ability to do activities of daily living. To the best of our knowledge, functional status as defined from form 2728 has not been validated, and remains another limitation of the administrative data. Comorbidities were also determined from form 2718, and even though it may be a better way to assess chronic conditions in ESRD patients, it was not possible to corroborate the information provided by the physician to patient-level information derived from medical records, and this validation gap remains another limitation. Additionally, individual level variables, including educational level and socioeconomic, that are not captured on form 2728 could be other predictors of interest and were not available for our analysis. Yet, by using the linked Medicare files, we were able to account for factors at the time of dialysis initiation as well as pre-dialysis health status in determining 1-year mortality.

Future directions include developing a decision making tool based on functional status and pre-dialysis health for patients initiating dialysis, which could guide both patients and physicians in shared decision making and conservative care options if appropriate. High quality studies are needed to examine comparative outcomes of conservative care and renal replacement modalities among elderly patients with poor functional status.

## Conclusion

Our findings indicate that poor functional status at the time of dialysis initiation is associated with significantly high 1-year mortality among elderly dialysis patients. Poor functional status also increases the risk of in-facility hemodialysis, and lack of arteriovenous access. Pre-dialysis health significantly impact clinical outcomes in elderly dialysis patients with poor functional status. In elderly patients with a large number of comorbidities, functional impairment and poor pre-dialysis health, a discussion regarding using conservative management or a time limited trial of hemodialysis may be appropriate. The information from the present study can influence health care provider’s decision to initiate dialysis, help counsel patients and families, and can facilitate an integrated approach in shared decision-making. Whether strategies to improve functional status in advanced stages of CKD would improve long-term clinical outcomes needs further investigation.

## Additional file

Additional file 1: Independent risk factors of mortality in incident end stage renal disease patients in the Cox proportional model. (DOCX 84 kb)

## Abbreviations

ASHD: Atherosclerotic heart disease
AV: Arteriovenous
CHF: Congestive heart failure
CI: Confidence interval
CKD: Chronic kidney disease
COPD: Chronic obstructive pulmonary disease
CVA/TIA: Cerebrovascular accident/transient ischemic attack
ESRD: End stage renal disease
GN: Glomerulonephritis
LVD: Large vessel disease
OR: Odds ratio
PD: Peritoneal dialysis
SD: Standard deviation
USRDS: United States renal data system
