# Supplementary material for: Functional status, pre-dialysis health and clinical outcomes among elderly dialysis patients
Source: BMC Nephrol. 2018 Apr 27;19:100. doi: 10.1186/s12882-018-0898-1 (PMC5924501; doi:10.1186/s12882-018-0898-1)
Supplement: Supplementary file 1 — Independent risk factors of mortality in incident end stage renal disease patients in the Cox proportional model. (DOCX 84 kb) [file 12882_2018_898_MOESM1_ESM.docx]

Additional file 1

Independent risk factors of mortality in incident end stage renal disease patients in the Cox proportional model

| Variable^a^ | Hazard Ratio |
| --- | --- |
| Poor functional status | 1.28 (1.24, 1.33) |
| Dialysis access  Central venous catheter  Peritoneal dialysis | 1.49 (1.43, 1.55)  1.27 (1.17, 1.37) |
| Age ≥ 80 years | 1.48 (1.44, 1.53) |
| Body mass index, kg/m^2^  <18.5  18.5-25  25-30  Missing | 1.62 (1.52, 1.72)  1.29 (1.25, 1.34)  1.10 (1.06, 1.33)  1.14 (1.03, 1.26) |
| Females | 0.95 (0.92, 0.97) |
| Race  Asians  Blacks  Hispanics  Native Americans | 0.70 (0.65, 0.76)  0.71 (0.69, 0.74)  0.71 (0.68, 0.74)  0.85 (0.74, 0.98) |
| History of nursing home | 1.44 (1.38, 1.49) |
| Unemployment | 1.01 (0.97, 1.06) |
| Comorbidities  Congestive heart failure  Atherosclerotic heart disease  Hypertension/large vessel disease  Diabetes melllitus  Cancer  Amputation  Peripheral vascular disease  CVA/TIA  Chronic obstructive pulmonary disease | 1.28 (1.25, 1.31)  1.09 (1.06, 1.11)  0.80 (0.77, 0.82)  1.02 (0.99, 1.05)  1.19 (1.15, 1.24)  1.03 (0.96, 1.10)  1.08 (1.05, 1.12)  1.04 (1.00, 1.08)  1.19 (1.15, 1.23) |
| Albumin, g/dL  <3.5  Missing | 1.42 (1.35, 1.50)  1.33 (1.25, 1.41) |
| Hemoglobin, g/dL  <11  11-12  Missing | 0.93 (0.87, 1.01)  0.90 (0.83, 0.99)  1.06 (0.96, 1.17) |
| Pre-dialysis acute hospitalization | 1.20 (1.15, 1.26) |
| Cause of ESRD  Cystic/hereditary  Glomerulonephritis  Hypertension/large vessel disease  Interstitial nephritis/pyelonephritis  Others/unknown  Malignancy  Vasculits/secondary GN | 0.75 (0.66, 0.86)  0.80 (0.75, 0.87)  1.04 (1.01, 1.08)  0.92 (0.85, 1.00)  1.08 (1.04, 1.14)  1.52 (1.41,1.63)  0.86 (0.76, 0.98) |
| Pre-dialysis nephrology care  0-12 months  >12 months  Unknown | 0.90 (0.87, 0.93)  0.83 (0.80, 0.86)  1.08 (1.04, 1.12) |
| Access to transplant information | 0.87 (0.85, 0.89) |

^a^Referents were good functional status to poor functional status, arteriovenous access for vascular access, > 30 for body mass index, male for females, White for race, no for comorbidities, >3.5 mg/dl for serum albumin, >12 for serum hemoglobin, , and diabetes mellitus for ESRD cause.

ESRD, end stage renal disease; CVA/TIA, cerebrovascular accident/transient ischemic attack; GN, glomerulonephritis
